# Development of behavioral patterns in young C57BL/6J mice: a home cage-based study

**DOI:** 10.1038/s41598-022-06395-1

**Published:** 2022-02-15

**Authors:** Maria Reiber, Ines Koska, Claudia Pace, Katharina Schönhoff, Lara von Schumann, Rupert Palme, Heidrun Potschka

**Affiliations:** 1 Institute of Pharmacology, Toxicology, and Pharmacy, Ludwig-Maximilians-University Munich, Koeniginstr. 16, 80539 Munich, Germany; 2 Department of Biomedical Sciences, Unit of Physiology, Pathophysiology and Experimental Endocrinology, University of Veterinary Medicine, Vienna, Austria; 3 Present Address: Fraunhofer Institute for Cell Therapy and Immunology IZI, Halle (Saale), Germany; 4 Present Address: Max Planck Institute of Biochemistry, Martinsried, Germany

**Keywords:** Developmental biology, Neuroscience

## Abstract

Evidence exists that behavioral patterns only stabilize once mice reach adulthood. Detailed information about the course of behavioral patterns is of particular relevance for neuroscientific research and for the assessment of cumulative severity in genetically modified mice. The analysis considered five age groups focusing on behavioral assessments in the animals’ familiar home cage environment during the adolescence phase. We confirmed age- and sex-specific differences for several of the behavioral parameters and fecal corticosterone metabolites. Interestingly, an age-dependent decline in saccharin preference was detected in female mice. Regardless of sex, relevant levels of burrowing activity were only observed during later developmental phases. The development of nest complexity following the offer of new material was affected by age in female mice. In female and male mice, an age-dependency was evident for wheel running reaching a peak at P 50. A progressive increase with age was also observed for Open field activity. The data sets provide guidance for behavioral studies and for development of composite measure schemes for evidence-based severity assessment in young mice. Except for the burrowing test, the different behavioral tests can be applied in different age groups during post-weaning development. However, age- and sex-specific characteristics need to be considered.

## Introduction

As a result of neuronal remodeling during the developmental trajectory of an individual, behavioral patterns as well as the response to potentially threatening situations and coping with distress can change tremendously during early development in mice^1–3^. Compelling evidence exists that behavioral patterns stabilize once the animals reach adulthood^1^. Various genetic mouse lines modeling neurodevelopmental and neuropsychiatric disorders display behavioral alterations during distinct developmental phases. These animal models can be characterized by peaks of distress during the ‘adolescence’ phase^4^. In neuroscientific preclinical research, genetically engineered mice provide powerful tools to investigate the linkages between genetic deficiencies and phenotypical alterations. In order to ensure a successful translation from preclinical model to clinical application, age- and sex-specific characteristics underlying the phenotypical profile of the genetic model have to be carefully considered. Thereby, robust and reproducible reference data^5^, rendering a ‘baseline behavioral profile’ in adolescent mice, can serve as a valuable basis to improve face validity in genetic animal models of neuropsychiatric disease.

Given the frequent use of the mouse as a model in animal-based research, it is of general interest and practical relevance to evaluate sensitive and conclusive parameters suitable for the assessment of severity in young mice. Parameters applied to assess the wellbeing of adult mice cannot merely be transferred as testing paradigms to mice undergoing different developmental stages. With a view to the current legal situation, the burden of genetically modified animals needs to be classified in three severity levels, in accordance with the EU Directive 2010/63/EU on the protection of animals used for scientific purpose^6^. As suggested by the German Centre for the Protection of Laboratory Animals (Bf3R), the classification is often based on two evaluations of the litter (newborn/weaning) including a clinical adspection of the offspring animals with assessment of the nutritional condition, body posture and body weight^7^. The next comprehensive clinical adspection and investigation of the individual animal is recommended when the animals reach an age of 2 months^7^. Thus, the phenotype of ‘adolescent’ animals is often not considered for the final assessment of genetically modified mouse lines. With regard to the cumulative burden experienced in the course of a lifetime, lacking information about the animal’s whole developmental period remains a particular cause of concern. Furthermore, the criteria currently applied for the assessment, based on clinical symptoms and alterations in activity^7^, might not comprehensively reflect the mouse’ wellbeing as a prey animal. In context of the 3R principle (Reduction, Replacement, Refinement), we aimed to develop sensitive approaches which can more precisely differentiate and grade different levels of distress, including more subtle alterations that remain undetectable with common clinical scoring systems. We assessed behavioral patterns that reflect the affective state of the animals, a range of non-essential activities as well as locomotor activity of the animals, to develop an adjusted composite measure scheme suitable for the assessment of severity in young mice. Aiming to reduce interference on the one hand, and to enable universal, standardized implementation of the respective parameters on the other hand, we focused on approaches applicable in the animal’s home cage environment. The applied candidate composite measure scheme was based on a comprehensive consortium data set for assessing and classifying severity in different adult rodent animal models^8^. Parameters with a high informative value were selected based on a bioinformatic approach among others comprising principal component analyses identifying the parameters best distinguishing between experimental and control animals and cross-correlation analyses guiding the selection of parameters with added value^9,10^.

We generated reference data in female and male C57BL/6JRj wildtype mice, one of the most commonly used background strains for genetically modified mice and the most widely used inbred mouse strain^11^, in order to assess the generalizability and robustness of selected parameters as a basis for candidate composite measure schemes in young mice. The comprehensive reference data are suitable for further subsequent testing in genetically modified mouse lines, and phenotypical characterization of the latter. Once validated in genetically altered strains, age-specific composite measure schemes provide a basis for evidence-based refinement recommendations in the sense of a reliable and comprehensive severity classification of genetic mouse models.

## Results

### Behavioral home cage assessment

#### Saccharin preference

**Figure 1:**
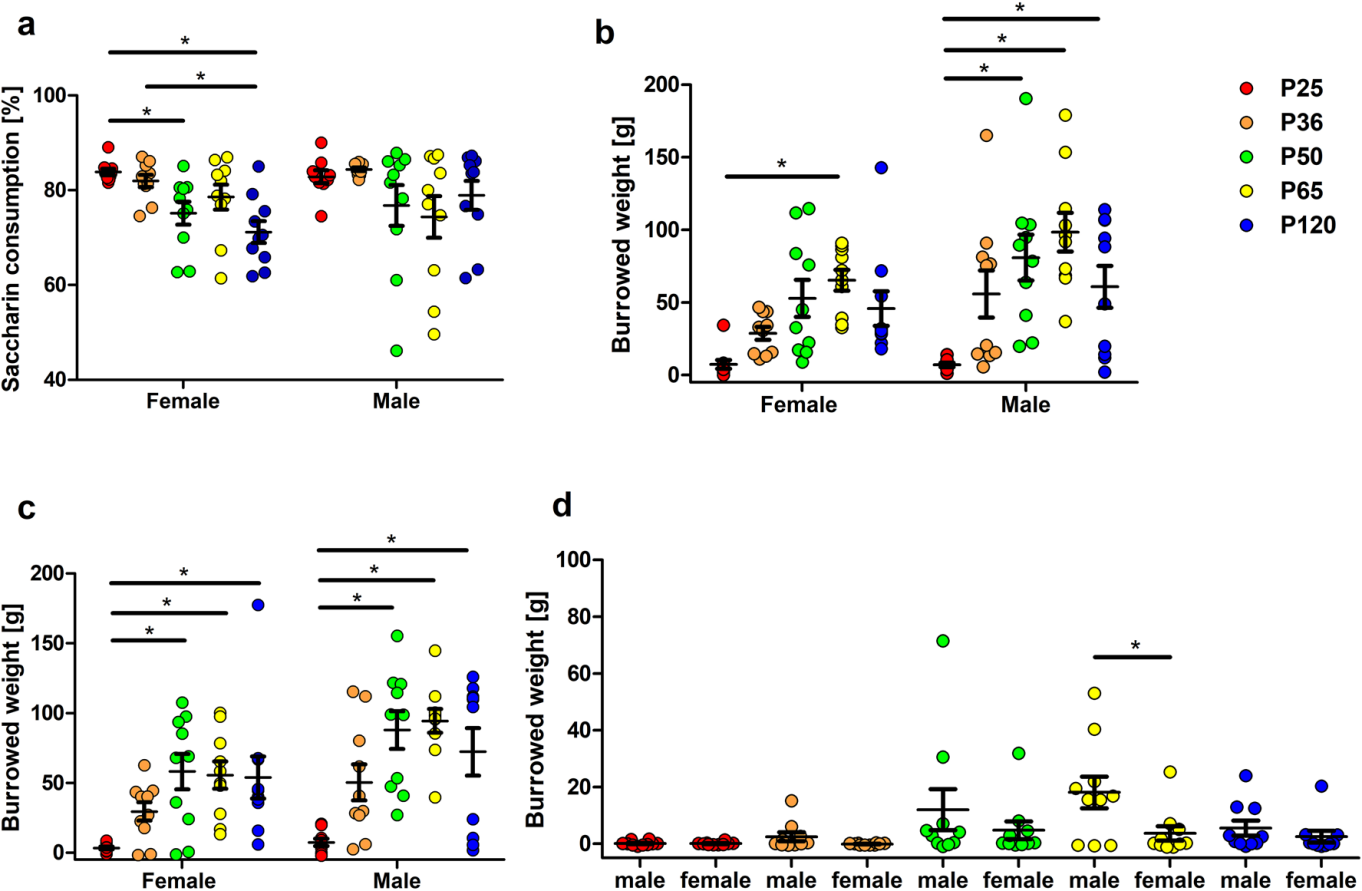
Saccharin preference and burrowing performance. (**a**) Age-dependent differences in saccharin preference, shown as total saccharin intake, were only detected in female mice: preference for saccharin in prepubescent and pubescent mice exceeded that of the adult control group, and prepubescent mice showed a higher preference than the respective sexually mature mice (F(4,45) = 6.495, p = 0.0003). Regardless of sex, burrowing performance overnight (first test) increased significantly in mice reaching sexual maturity (**b**, first test; interaction p = 0.5912, age phase p < 0.0001, sex p = 0.0051) (**c**, second test; interaction p = 0.6044, age phase p < 0.0001, sex p < 0.0020). A difference between sexes was only detected in the second burrowing test during the light phase in young adult animals (**d**) (interaction p = 0.2394, age phase p = 0.0068, sex p = 0.0119). One-way ANOVA, followed by Bonferroni multiple comparison tests for (**a**), and two-way ANOVA, followed by Bonferroni multiple comparison tests for (**b**–**d**). *p < 0.05. Colored dots refer to the respective age groups. Error bars indicate the standard error of the mean (SEM).

All animal groups displayed a preference for sweetened saccharin solution (Fig. 1a). The analysis of saccharin preference at different time points during the post-weaning phase revealed an age-dependency with young female animals exhibiting a stronger preference for the sweet solution (Fig. 1a). Both, prepubescent (P25) and pubescent (P36) female mice consumed a higher amount of saccharin solution in comparison to mature adult (P120) female mice (Fig. 1a, F(4,45) = 6.495, p = 0.0003). The statistical differences between prepubescent and sexually mature (P50) mice indicate a decline in saccharin preference, when female animals reach sexual maturity (Fig. 1a, F(4,45) = 6.495, p = 0.0003). In contrast to female mice, the respective analysis of saccharin preference in male mice did not identify significant age-dependent differences (Fig. 1a, F(4,44) = 1.694, p = 0.1686). However, in both, female and male mice the variance increased from P50 onwards (Fig. 1a), indicating more pronounced interindividual differences as compared to younger animals.

#### Burrowing

The overnight burrowing performance developed with increasing age in female and male mice (Fig. 1b,c). Concerning the overnight (dark phase) burrowing activity, at P25 no relevant burrowing activity was detected in animals of both sexes during the two tests (Fig. 1b, interaction p = 0.5912, age phase p < 0.0001, sex p = 0.0051; Fig. 1c, interaction p = 0.6044, age phase p < 0.0001, sex p = 0.0020). At P36 interindividual differences were increasing with some male animals already exhibiting a higher level of burrowing (Fig. 1b,c). However, a significant increase as compared to P25 was only confirmed from P50 onwards. This increase became evident in both female and male animals. Comparison of the findings from both test days did not indicate pronounced differences (Fig. 1b,c).

While no sex differences were detected for overnight (dark phase) burrowing activity (Fig. 1b,c), young adult (P65) male mice displayed a higher burrowing performance than the respective female mice during the 120 min test session (light phase) (Fig. 1d, interaction p = 0.2394, age phase p = 0.0068, sex p = 0.0119). However, this difference became only evident during the second of the two burrowing test days. The analysis of burrowing activity during the 120 min test sessions did not reveal age-dependent differences with a relatively low mean performance in almost all age groups (Fig. 1d, Supplementary Fig. S1a).

#### Nest building

**Figure 2:**
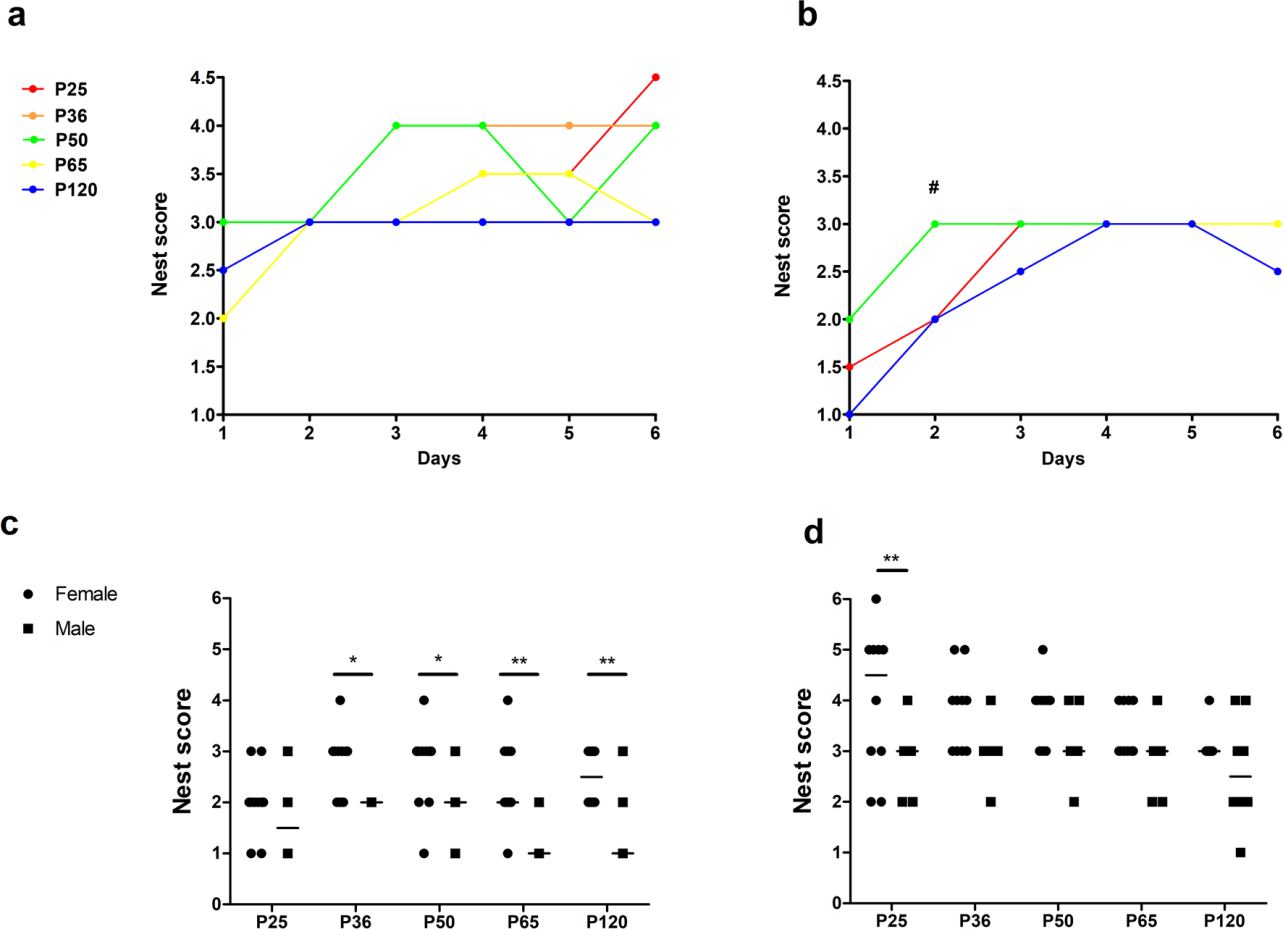
Nest building performance and nest complexity. The development of nest complexity scores differed between age phases in (**a**) female animals (F(4, 290) = 2.75991, p = 0.02804), but not in (**b**) male animals (F(4, 290) = 1.07471, p = 0.3692). In male animals, the nest scores in P50 animals exceeded that of P120 animals on the second day (**b**, #) (F(4, 45) = 2.885, p = 0.0329). Sex differences were evident (**c**) on the first day in all age groups except for P25 animals (interaction p = 0.3161, age phase p = 0.0205, sex p < 0.0001). In contrast, sex differences in P25 mice became evident (**d**) only on the last day (interaction p = 0.6905, age phase p = 0.0504, sex p < 0.0001). Linear regression for (**a**) and (**b**); one-way ANOVA, followed by Bonferroni multiple comparison tests for #; two-way ANOVA followed by Bonferroni multiple comparison tests for (**c**) and (**d**), *p < 0.05, median. Colored dots/lines refer to the respective age groups.

Concerning the development of the nest complexity scores over the total observation period of 6 days, age-dependent differences in the slope of the nest complexity curve were observed in female (Fig. 2a, F(4, 290) = 2.75991, p = 0.02804) but not in male mice (Fig. 2b, F(4, 290) = 1.07471, p = 0.3692). When directly comparing scores from different days of the observation phase among the age groups, we only identified a significant difference at day 2 with nest building performance of male mice aged 50 days exceeding that of adult mice (Fig. 2b, F(4, 45) = 2.885, p = 0.0329).

From P36 onwards, sex differences were always evident at the first day of the observation period (Fig. 2c, interaction p = 0.3161, age phase p = 0.0205, sex p < 0.0001). These data indicate a longer latency to construct complex nests in male mice. Differences between female and male mice were also confirmed by mean sum scores from day 1 to 6, and on other selected observation days at different age levels (Fig. 2d, interaction p = 0.6905, age phase p = 0.0504, sex p < 0.0001, Supplementary Fig. S2a–d). Thereby, female mice exhibited a better nest building performance than male mice.

#### Voluntary wheel running

**Figure 3:**
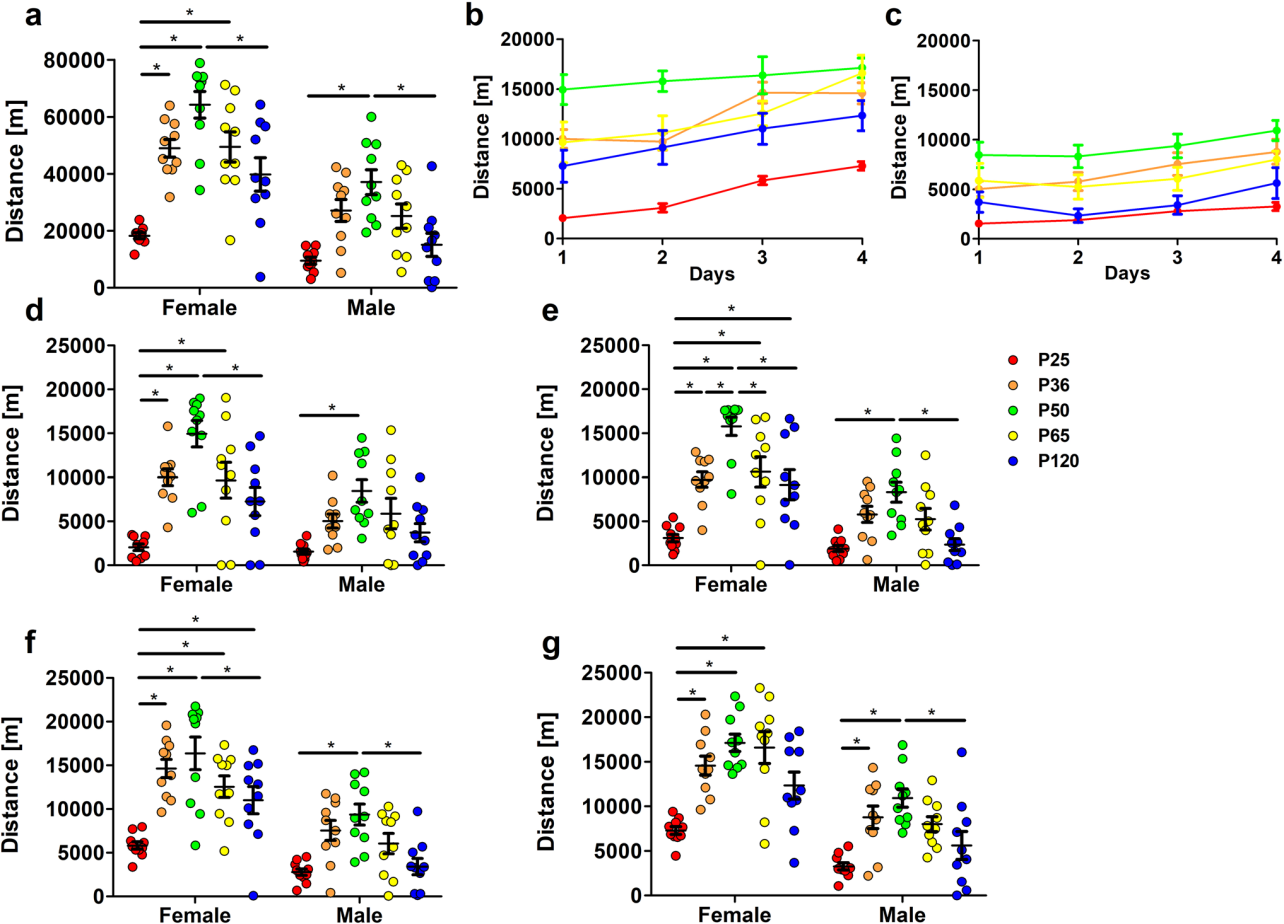
Voluntary wheel running. The total distance moved during the entire observation period (**a**) increased until animals reached sexual maturity (interaction p = 0.1802, age phase p < 0.0001, sex p < 0.0001). Analysis of the increase in activity during the testing phase did not reveal age-related differences in female (**b**) (F(4,190) = 1.02147, p = 0.3974) and male animals (**c**) (F(4,190) = 0.367026, p = 0.8319). Assessment of running activity at separate days (day 1–4) (**d**–**g**) demonstrated that running activity in P50 animals exceeded that of the sex-matched P25 group on all test days. Two-way ANOVA followed by Bonferroni multiple comparison tests for (**a**,**d**–**g**); linear regression for (**b**) and (**c**); *p < 0.05. Colored dots/lines refer to the respective age groups. Error bars indicate the standard error of the mean (SEM).

Considering activity over the course of the four testing days, animals of all age phases and of both sexes performed wheel running. In female and male mice, the total distance moved in the wheel during the 4 days of testing significantly increased during the weeks after weaning, reaching a peak at P50 (Fig. 3a, interaction p = 0.1802, age phase p < 0.0001, sex p < 0.0001).

A decline became significantly evident in female and male mice with further development when comparing P50 and P120 data (Fig. 3a).

Sex differences were detected in all age groups except for the group of prepubescent mice (Fig. 3a). The distance moved in female mice exceeded that in male mice from pubescent age onwards (Fig. 3a). Considering the increase in activity over the course of the four testing days, we did not detect any differences in all groups of both sexes (Fig. 3b,c, females F(4,190) = 1.02147, p = 0.3974, males F(4,190) = 0.367026, p = 0.8319). Hence, the distance moved per day did not increase significantly during the test phase in all groups. A breakdown of running activity per day reveals that the activity level in P50 animals of both sexes was increased on each day of testing as compared to the respective P25 group (Fig. 3d,e–g).

### Open field test

The first minutes in a novel environment can provide information about exploratory activity.

**Figure 4:**
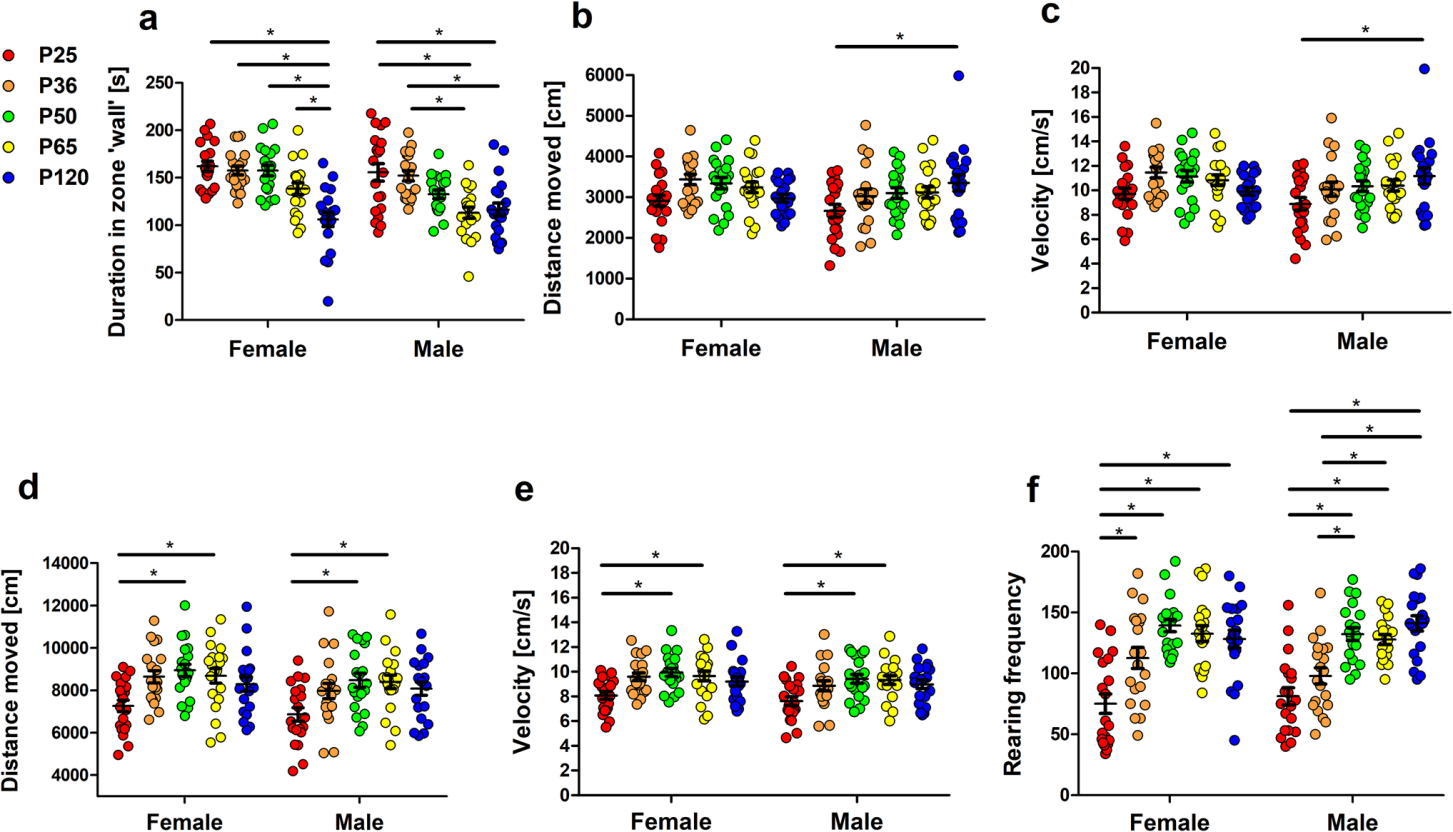
Open field test. In the first 5 min of the Open field test, illustrated in (**a**–**c**), prepubescent and pubescent animals of both sexes spent more time in the ‘wall’ zone than adult mice (**a**) (interaction p = 0.0239, age phase p < 0.0001, sex p < 0.0095). Male prepubescent animals moved a shorter distance (**b**) (interaction p = 0.0733, age phase p = 0.0122, sex p = 0.1613) and moved more slowly (**c**) (interaction p = 0.0734, age phase p = 0.0125, sex p = 0.1618) than the sex-matched adult group. Analysis of activity during the total test duration of 15 min, illustrated in (**d**–**f**), revealed that the distance moved (**d**) and velocity (**e**) in P50 and P65 mice exceeded that of the respective prepubescent animals (**d**, interaction p = 0.9626, age phase p < 0.0001, sex p = 0.0441, **e**, interaction p = 0.9628, age phase p < 0.0001, sex p = 0.0440). Independent of the sex of the animals, rearing frequency (**f**) proved to be lower in prepubescent animals as compared to sexually mature, young adult, and adult animals (interaction p = 0.2458, age phase p < 0.0001, sex p = 0.7122). Two-way ANOVA, followed by Bonferroni multiple comparison tests, *p < 0.05. Colored dots refer to the respective age groups. Error bars indicate the standard error of the mean (SEM).

During the first 5 min in the Open field, prepubescent and pubescent animals of both sexes spent more time in the ‘wall’ zone than adult mice (Fig. 4a, interaction p = 0.0239, age phase p < 0.0001, sex p < 0.0095). Thereby, sex differences became evident in the groups of P50 and P65 mice with females spending longer times in the ‘wall’ zone than age-matched male mice (Fig. 4a). In this early phase, male prepubescent animals moved a shorter distance (Fig. 4b, interaction p = 0.0733, age phase p = 0.0122, sex p = 0.1613) and moved with lower speed (Fig. 4c, interaction p = 0.0734, age phase p = 0.0125, sex p = 0.1618) in comparison to male adult animals.

Data from the total monitoring phase of 15 min can inform about the overall activity status and locomotion. Distance moved and velocity in P50 and P65 mice exceeded that in respective prepubescent animals (Fig. 4d, interaction p = 0.9626, age phase p < 0.0001, sex p = 0.0441, Fig. 4e, interaction p = 0.9628, age phase p < 0.0001, sex p = 0.0440). Independent of the sex of the animals, rearing frequency proved to be lower in prepubescent animals as compared to sexually mature, young adult and adult animals (Fig. 4f, interaction p = 0.2458, age phase p < 0.0001, sex p = 0.7122). In female animals, the age-dependent increase was already evident at an earlier time point during development with a significant difference between prepubescent and pubescent animals (Fig. 4f). However, comparison of rearing data within the different age groups did not indicate relevant sex differences.

### Irwin score

**Figure 5:**
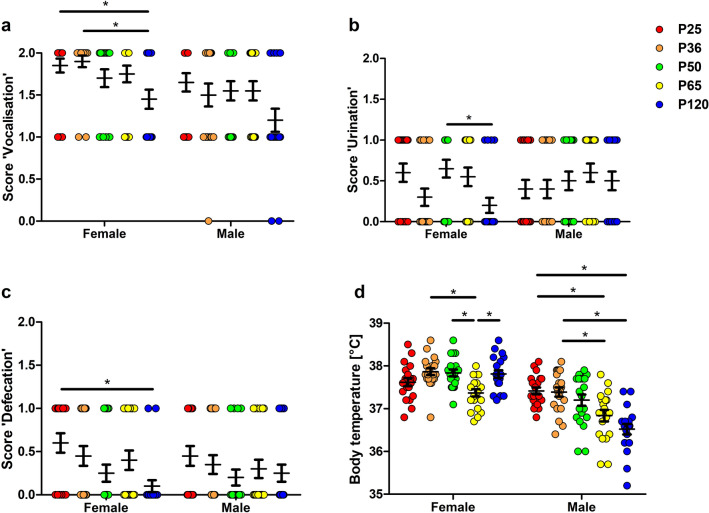
Irwin score. As compared to female adult animals, handling-associated vocalization (**a**) and defecation (**c**) scores were increased in prepubescent animals. Handling-associated urination (**b**) in sexually mature female animals exceeded that of female adult animals (**a**, F(4,95) = 3.387, p = 0.0123; **b**, F(4,95) = 3.437, p = 0.0114; **c**, F(4,95) = 3.474, p = 0.0108). Rectal body temperatures (**d**) in female mice exceeded those in male mice in all age groups except for P25, and body temperatures in pubescent animals of both sexes were higher as compared to those in young adult animals (interaction p < 0.0001, age phase p < 0.0001, sex p < 0.0001). One-way ANOVA, followed by Bonferroni multiple comparison tests for (**a**–**c**), and two-way ANOVA, followed by Bonferroni multiple comparison tests for (**d**); *p < 0.05. Colored dots refer to the respective age groups. Error bars indicate the standard error of the mean (SEM).

The analysis of the Irwin observation test, a widely applied neurobehavioral assay, revealed several age-specific characteristics of handling-associated parameters. While Irwin sum scores did not indicate sex- or age-dependent differences (Supplementary Fig. S3a), we detected an age-dependency of selected Irwin parameters in female mice (Fig. 5a–d). The scores for handling-associated vocalization were higher in prepubescent and pubescent female animals compared to female adult animals (Fig. 5a, F(4,95) = 3.387, p = 0.0123). Moreover, the scores for handling-associated urination in female sexually mature animals exceeded those in female adult animals (Fig. 5b, F(4,95) = 3.437, p = 0.0114). Handling-associated defecation was more pronounced in prepubescent female mice than in female adult mice (Fig. 5c, F(4,95) = 3.474, p = 0.0108). The comparison of Irwin data between female and male animals within the different age groups did not confirm relevant sex differences. The only exception were rectal body temperatures in female mice, which exceeded those in male mice in all age groups from P36 onwards (Fig. 5d, interaction p < 0.0001, age phase p < 0.0001, sex p < 0.0001). An age-dependency of body temperature was confirmed in both sexes with a higher temperature in female and male pubescent mice than in young adult mice (Fig. 5d). Moreover, the rectal temperature in prepubescent male mice exceeded that in young adult and adult animals (Fig. 5d).

### Fecal corticosterone metabolites

**Figure 6:**
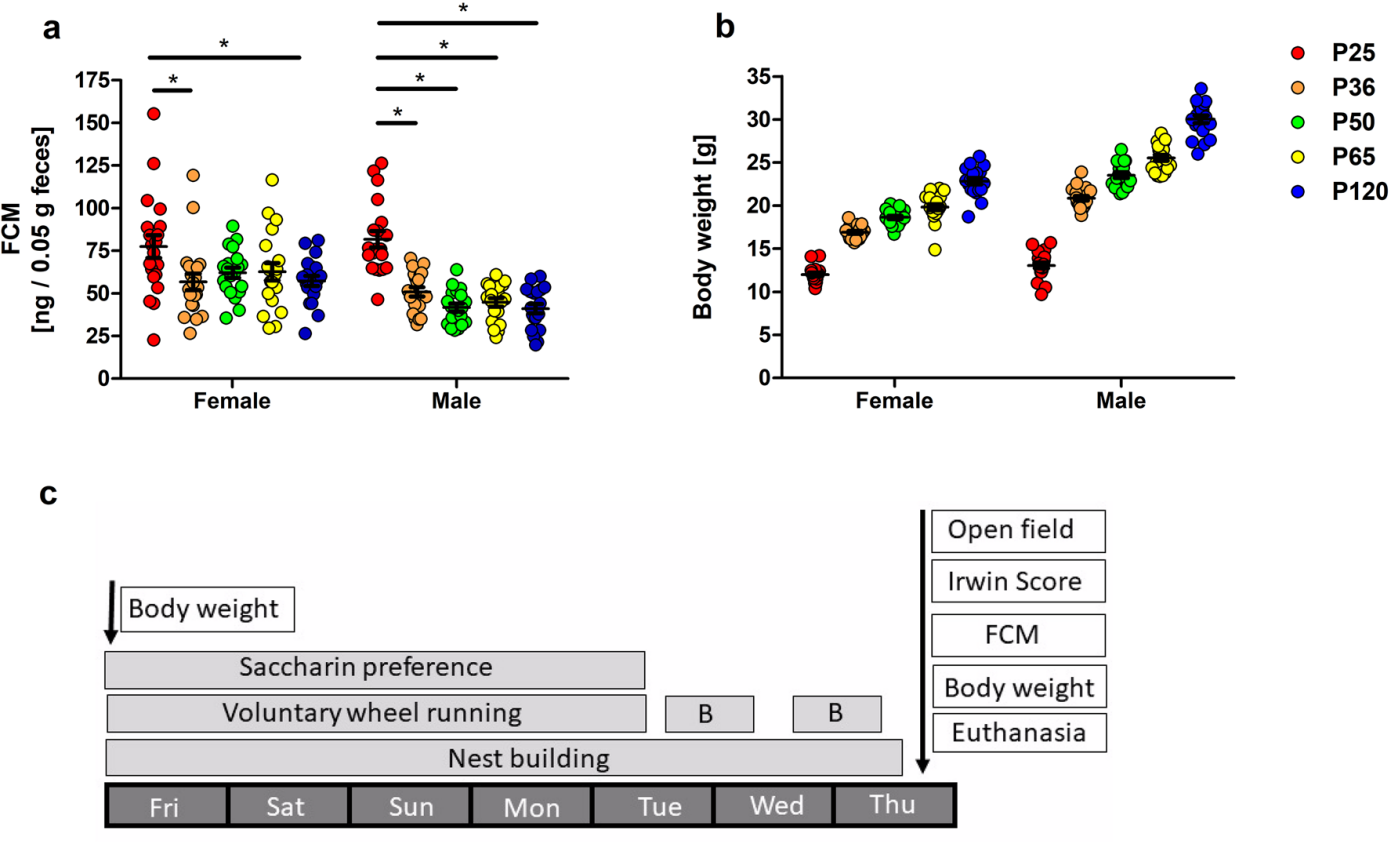
Fecal corticosterone metabolite concentrations (FCM) (**a**) were higher in prepubescence than in pubescence and adulthood in both female and male animals (interaction p = 0.0130, age phase p < 0.0001, sex p < 0.0001). Moreover, concentrations in prepubescent male animals were significantly higher than in all other age groups. Two-way ANOVA, followed by Bonferroni multiple comparison tests, *p < 0.05. Error bars indicate the standard error of the mean (SEM). The age-dependent body weight increase in the different age groups is illustrated in (**b**). Colored dots refer to the respective age groups. (**c**) Illustrates an overview of the weekly experimental scheme. *B* burrowing.

In prepubescent male animals, the concentration of fecal corticosterone metabolites was significantly higher as compared to all other age groups (Fig. 6a, interaction p = 0.0130, age phase p < 0.0001, sex p < 0.0001). In female animals, the concentrations during prepubescence exceeded those from pubescent and adult animals (Fig. 6a, interaction p = 0.0130, age phase p < 0.0001, sex p < 0.0001). We did not detect sex differences within the age groups.

### Body weight

When comparing data from the beginning and end of the different testing phases in all age groups, a pronounced body weight gain was only evident for the groups of prepubescent female and male mice (Supplementary Fig. S3b). As expected an age-dependent body weight increase was confirmed when comparing the different age groups (Fig. 6b).

## Discussion

In line with our aim, this study identified significant age-dependent differences in the development of behavioral patterns in the early developmental phase following weaning. Moreover, sex was confirmed as an influencing factor, with differences evident for the development of selected parameters. The data sets provide comprehensive information for behavioral studies in neuroscience and for the development of age-specific composite measure schemes for evidence-based severity assessment approaches.

The parameters of interest have been selected based on consortium data, which were obtained in adult rodent disease models, and which supported an informative value for the purpose of severity assessment (e.g.^8,9,12–20^). Information about model-specific characteristics provide key elements for an evidence-based grading of severity, and allow filling of gaps in model-specific refinement recommendations^8^. Based on the promising candidate parameters, fully validated in adult rodent models (e.g.^9,12–15,17^), we subsequently tested for applicability in young mice. Moreover, the compilation of tests considered aspects of animal welfare such as the ethical necessity of group housing, and also allows practical implication in the majority of conventional animal facilities. Behavioral tests conducted in the home cage under standardized housing conditions allow phenotypical profiling throughout the circadian rhythm, thereby minimizing external influences and factors of distress experienced by the prey animals^21,22^. Sensitive home cage measures allow depiction of even subtle and spontaneous changes of behavioral patterns, and standardized housing strengthens reproducibility and inter-facility comparability of preclinical data^5^.

To our knowledge, a study in the rat kindling paradigm was the first exploring saccharin preference as a potential parameter for severity assessment^15^. In subsequent studies, we have detected an influence of different chronic models of epilepsy^13,14,23^. Assessment of the preference for a sweet solution has originally been integrated as a behavioral test in research focusing on psychiatric disorder and depression-associated alterations in laboratory rodents (e.g.^24–26^). Thereby, reduction or loss of the preference for a sweet solution is interpreted as sign of anhedonia-associated behavior, i.e. the reduction or loss of the ability to experience pleasure. A recent study evaluating the depression-like phenotype of different mouse strains in animals aged 22–26 days and 32–36 days reported significant differences between the strains in the preference for sucrose during adolescence^27^. An age-dependency of saccharin preference has been previously described in groups of female and male rabbits. Surprisingly, younger animals aged between 4 and 6 months preferred water, whereas older rabbits aged between 31 and 69 months preferred the sweet solution^28^. In contrast, we demonstrated an age-dependency of saccharin preference in female mice with higher levels of saccharin consumption in prepubescent and pubescent mice, and a significant decline already evident at P50, when animals reach sexual maturity. Interestingly, there was a lack of differences between age groups in male mice. The fact that we confirmed a relevant saccharin preference in all age groups in both sexes suggests that saccharin preference can be applied in young animals even in the early phase following weaning. Interestingly, we confirmed lower interindividual variance of the preference for sweetness in young female and male wildtypes. Considering the typically increased behavioral variability displayed by young animals during their post-weaning development, these findings might reinforce the particular suitability of the saccharin preference test, even suggesting that lower animal numbers might be necessary to reach a sufficient test-specific power. Concerning the value as a severity assessment parameter in young mice, future studies need to address the impact of intervention- or model-associated distress on the consumption ratios.

Burrowing and nest building activity have been suggested as valuable indicators of distress, pain, suffering and a compromised well-being in mice^29^. Both behavioral patterns are considered non-essential but evolutionary preserved^29^. Burrowing activity proved to serve as a sensitive indicator of well-being compromised by painful procedures, sickness behavior associated with immune system activation and models of psychiatric or neurological disorders^29–32^. Overnight burrowing performance developed with increasing age in young mice reaching a relevant activity at P50 in both sexes. The 120 min assessment towards the end of the light phase did not confirm a relevant burrowing performance during this time phase. This might be related to the fact that the exposure was limited to two test days without a longer habituation phase in advance. In contrast, data from the overnight phase suggest that burrowing activity can be applied as a severity assessment parameter in female and male mice aged 50 days and beyond, i.e. when animals reach sexual maturity. However, one needs to pay attention to the high level of variance, which needs to be considered when determining the necessary animal number. Moreover, follow-up studies are necessary—as for all other parameters of interest—to determine the sensitivity to experimental distress. In this context, two studies are of interest, which reported a higher sensitivity of burrowing performance in 19-months-old or 21-months-old mice to the influence of exposure to a viral mimetic or to lipopolysaccharide, when compared to the response of 4-months-old mice^33,34^. However, these results might rather be related to a more pronounced sickness response in aged animals than to a difference in the sensitivity of burrowing activity towards stressors.

To our knowledge the only other study that evaluated burrowing in young mice focused on mice aged 31 days. Regardless of the strain the authors reported that mice at this age exhibit burrowing activity^35^. In line with our data, the authors did not report sex differences.

Concerning our findings in prepubescent and pubescent mice, it might be of future interest to assess the influence of the burrowing material, as the body size in relation to the particle size of the burrowing material might matter in very young animals. In this context, it is of interest that the choice of the burrowing material affected the sensitivity of burrowing as a parameter for pain assessment in a multicenter study in rats^36^.

The pronounced sensitivity of nest building to detrimental influences has for instance been demonstrated as a consequence of abdominal surgery, transmitter implants, systemic inflammation, hippocampal scrapie infection, models of psychiatric disorders, and Alzheimer models^30,37–43^. Based on studies exploring the influence of pain on nest building performance, Turner and colleagues^44^ suggested analysis of nest building behavior as an important parameter for pain assessment. Earlier reports from adult mice reported that nest building performance in female mice exceeds that in male mice^45^. In line with these findings from adult animals, the data sets from young female mice also confirm a higher rate of nest building with a reduced latency to construct complex nests.

An impact of age on nest building performance has previously been described in a study focused on the impact of ageing reporting a decline in nest building performance in 25-month-old mice as compared to 7-month-old mice^45^. This study raises the question, if there is also an impact of age in phases of early post-weaning development. While only minor age-dependent differences were detected in male mice in our study, the analysis of the slope of nest complexity curves from female mice revealed age-dependent differences in the latency to construct complex nests. Thereby, the steeper curve in prepubescent female mice suggests a longer latency in younger animals. The findings confirm that a relevant nest building activity can already be observed in young female and male mice. This is in line with an earlier report by Moy and colleagues^46^ describing relevant nest building in different mouse strains at an age of 3–4 weeks.

The development of nest complexity during the 6-day observation phase in our study suggests that an influence of experiment-associated distress should rather be evaluated towards end of the week before replacing the nest material by a new batch. It is likely that the application of nest complexity as a severity assessment parameter needs to consider various influencing factors including the mouse strain, ambient temperature, and nest material as these have been reported to exert tremendous effects on the performance in adult^47–49^ and young mice^35,46^. Respective factors might have also contributed to the very low interest to construct complex nests observed in P30 and P40 mice in an earlier study^35^.

Häger and colleagues^50^ have analyzed the informative value of voluntary wheel running as a basis for observer-independent fine-scale grading of severity levels. In their initial study, the group confirmed a negative influence of subchronic restraint stress and a colitis model^50^. In subsequent studies, the sensitivity of voluntary wheel running to recurrent stress exposure and a dextran sodium sulphate-induced colitis model was further confirmed^17,18^. Based on these findings, it is of particular interest to assess the development of voluntary wheel running performance in young mice as this might provide a basis for the application of this sensitive severity assessment parameter in earlier developmental phases. Comparison between age groups of young mice revealed a progressive increase during the early post-weaning phase reaching a peak at P50, i.e. a time point at which animals are considered sexually mature. Interestingly from P36 onwards a pronounced sex difference was evident with voluntary wheel running activity in female mice exceeding that in male mice. Nevertheless, in both sexes a relevant activity was observed at different age levels. The development on subsequent days suggests that habituation is of relevance in very young animals and that assessment should focus on activity following at least three habituation days. However, at the earliest age assessed in the present study, we cannot exclude that the development of the performance at subsequent testing days was also influenced by the growth curves and development of muscle strength of the young animals. At later age levels, we did not detect a relevant habituation impact, so that assessment can be based on data from the first days of wheel exposure.

The age-dependent development of activity patterns has already been assessed in mice in various previous studies^1,35,51–54^. These studies demonstrated a progressive increase of activity levels during the developmental phase that corresponds to adolescence in humans. In this context, some groups have also applied the Open field paradigm, which can provide information about exploratory and locomotor behavior and other activity patterns such as rearing^24^. In line with available data, we confirmed a progressive increase of velocity and distance moved as well as rearing frequency reaching a plateau from P50 onwards without any further age-dependent differences from this time point on. The analysis of exploratory behavior during the first 5 min of the paradigm revealed a lower locomotion level in prepubescent female and male mice in comparison with selected other age groups. More importantly, these very young animals exhibited a higher level of thigmotaxis spending more time in the wall zone of the Open field. Thigmotaxis proved to be more pronounced in female mice than male mice. While we did not design the Open field as a test for anxiety-associated behavior in this study and applied rather dim light conditions allowing to focus on locomotion and activity patterns, these data suggest a higher level of anxiety-associated behavior in this early post-weaning phase. In previous studies focusing on the development of anxiety-associated behavior in young mice, controversial data have been reported^1,35,51–53^. This might be related to the influence of various factors including environmental conditions, maternal care, handling procedures, habituation to the laboratory and to the person handling the animal during the experiment.

Our findings from the Open field suggest that the paradigm might be applied at different age levels in young mice. However, the data emphasize the need to consider the characteristic behavioral patterns at different age levels. Moreover, it seems to be of particular relevance to pay attention to the course of behavioral patterns during the test time with a separate analysis of different time bins.

Irwin scores have been developed for the assessment of the adverse effect potential of central nervous system (CNS) drug candidates^55^. The parameters have been selected to provide information about the autonomic, peripheral and central nervous system and behavioral patterns. We have recently developed an adjusted Irwin score excluding all parameters that intensely interfere with the animal in order to avoid any stress bias related to the assessment. This adjusted Irwin score was successfully applied in different models. It proved to provide valuable information about the well-being and general condition in the early post-surgical phase and following the induction of a status epilepticus^13,14,56^. While the Irwin sum scores were in a comparable range, the analysis of selected parameters revealed an age-dependency. These parameters comprised vocalization and handling-associated defecation reaching higher scores in younger female mice as well as handling-associated urination with a peak in female mice at an age of 50 days. As expected higher rectal body temperatures were confirmed in younger mice of both sexes. In this context, we cannot exclude a bias by an age-dependent difference in the response to the handling procedure necessary to determine rectal body temperature. Taken together, application of an Irwin scoring system for the purpose of severity assessment in young mice needs to consider the age-specific characteristics in these selected parameters.

The measurement of fecal corticosterone metabolites serves as a non-invasive parameter for assessing adrenocortical activity (4–12 h prior to feces sampling in mice) and has been applied in a range of species including laboratory rodents^57,58^. To our knowledge, age-related differences have been rarely studied and reported in mice. One longitudinal study in females of two mouse strains reported age- and strain-related differences^59^. The data obtained in our experiments revealed an age-dependency of the fecal corticosterone metabolite levels with prepubescent animals of both sexes showing higher baseline levels as compared to the mature adult control group. However, external influences such as the more recently experienced procedure of weaning, separation from the litter, and new housing conditions, have to be considered as factors in the youngest age group, which may contribute to increased levels of distress^60^. Although sex differences in excreted corticosterone metabolites (lower levels in males) have been reported^58,60,61^, they were not significant in our study.

The analysis of baseline performance in the different age phases benefits from behavioral assessments in the animals’ home cages since respective alterations in the phenotypical profile during post-weaning development draw a subtler and more precise picture of observer-independent and spontaneous characteristics. As already emphasized follow-up studies are necessary to assess the sensitivity of the parameters to distress in the different age levels. Moreover, the impact of various factors needs to be explored in mice of different age. Among others, these include handling procedures, habituation procedures, environmental factors, and time point of weaning. In this context, the sequence and parallel analysis of different parameters also needs to be taken into account, when drawing conclusions and when designing future studies further exploring the generalizability and robustness of the findings from this study.

In conclusion, the data sets provide valuable guidance for behavioral studies in young mice and for the development of composite measure schemes for evidence-based severity assessment in young mice. The majority of the behavioral tests can be applied in different age groups during early post-weaning development. However, age- and sex-specific characteristics need to be considered. As an exception the burrowing paradigm should only be used in mice with an age of at least 50 days.

## Materials and methods

### Ethical statement

All animal experiments were conducted and reported in accordance with the EU Directive 2010/63/EU and the German Animal Welfare Act. All investigations were carried out in line with the ARRIVE (Animal Research: Reporting of In Vivo Experiments) guidelines and the Basel declaration (http://www.basel.declaration.org) including the 3R principle. All animal experiments were approved by the government of Upper Bavaria (Munich, Germany, license number ROB-55.2-2532.Vet_02-19-157). As requested by the government of Upper Bavaria, the required sample size had been calculated by a power analysis before the start of the study.

### Animals

Pregnant C57BL/6JRj mice (n = 51), obtained at embryonic stages (E) 10, E11 and E13 (Janvier Labs, Le Genest-Saint-Isle, France), were single-housed under controlled environmental conditions (22–24 °C, 45–60% humidity) in individually ventilated cages (Tecniplast Deutschland GmbH, Hohenpeißenberg, Germany) in a 12-h dark–light cycle with ad libitum access to food (Ssniff Spezialdiäten GmbH, Soest, Germany) and tap water. Each cage was supplemented with bedding material (Lignocel Select, J. Rettenmaier & Söhne GmbH & Co. KG, Rosenberg, Germany), 14 g of nesting material (Enviro Dri, Claus GmbH, Limburgerhof, Germany), a wood brick (Labodia AG, Niederglatt, Switzerland), and a triangular mouse house (Zoonlab GmbH, Castrop-Rauxel, Germany). Mother animals with their litters obtained a fresh cage at postnatal day (P) 6 and 14. From weaning at P21 until the start of the experiments, experimental animals (n = 200, 50% female/male), C57BL/6JRj offspring mice, were randomly divided (http://www.randomizer.org) in experimental units of same-sex siblings (n = 2) and each experimental unit was housed in a Makrolon cage type III (Ehret GmbH & Co. KG, Emmendingen, Germany), providing bedding material (Lignocel Select, J. Rettenmaier & Söhne GmbH & Co. KG, Rosenberg, Germany), two nestlets (Ancare, Bellmore, New York, USA), and one square animal house (Zoonlab GmbH, Castrop-Rauxel, Germany). The order of cages was randomized (http://www.randomizer.org). Animals received a fresh cage once per week on Tuesdays. During the observation period, animals were housed in groups of two as the respective experimental unit in a home cage system with continuous video recordings (PhenoTyper, Noldus, Wageningen, the Netherlands). Each PhenoTyper was supplemented with 200 g bedding material (Lignocel Select, J. Rettenmaier & Söhne GmbH & Co. KG, Rosenberg, Germany), two nestlets (Ancare, Bellmore, New York, USA), an infrared translucent shelter (Noldus, Wageningen, the Netherlands) and two drinking bottles (Noldus, Wageningen, the Netherlands). The home cage system provided a removable wall for the insertion of a running wheel (PhenoWheel, Noldus, Wageningen, the Netherlands).

### Experimental design

In the present study, offspring animals were randomly (http://www.randomizer.org) distributed to the following four postnatal time phases (Table 1), representing the different developmental stages in murine lifetime^1^: The ‘adolescence’ phase: (1) prepubescent, experiments starting at P25 (no age range), (2) pubescent, experiments starting at P36 (age range: P35–36), and (3) sexually mature mice, experiments starting at P50 (no age range); followed by (4) young adult mice, experiments starting at P65 (no age range). A fifth group of mature adult mice from P120 onwards (experiments starting at P120, age range: P117–120), representing a frequently used age in experimental mice, was additionally tested. Experimental animals (n = 200) of all five age phases (n = 40 per age phase) and of either sex (n = 20 per age phase and sex) were investigated. For the tests conducted in the PhenoTyper home cage, the analysis was performed per experimental unit (n = 2). All behavioral tests carried out in the PhenoTyper home cage were video recorded. As a measure against risk of bias and with regard to the weekly workflow in the animal facility and its possible impact on animal’s behavior on certain days, experimental testing for all groups was based on a weekly routine (Fig. 6c). Experiments were executed in four batches per age phase, resulting in a total duration of 20 weeks.

**Table 1:** Group allocation and age of experimental animals undergoing the behavioral test battery. *SP* saccharin preference, *NEST* nest building performance, *VWR* voluntary wheel running, *BUR* burrowing, *OF* open field, *IRW* Irwin score, *FCM* fecal corticosterone metabolites, *BW* body weight.

Group	Represented age phase	n female/male	Behavioral tests (observation period)	Age range in days
P25	Prepubescence	20/20	SP, NEST, VWR (P25-P29)	± 0
			BUR (P29 and P30)	
			OF, IRW, FCM (P31)	
			BW (P25 and P31)	
P36	Pubescence	20/20	SP, NEST, VWR (P36-P40)	− 1
			BUR (P40 and P41)	
			OF, IRW, FCM (P42)	
			BW (P36 and P42)	
P50	Sexual maturity	20/20	SP, NEST, VWR (P50-P54)	± 0
			BUR (P54 and P55)	
			OF, IRW, FCM (P56)	
			BW (P50 and P56)	
P65	Young adulthood	20/20	SP, NEST, VWR (P65-P69)	± 0
			BUR (P69 and P70)	
			OF, IRW, FCM (P71)	
			BW (P65 and P71)	
P120	Mature adulthood	20/20	SP, NEST, VWR (P120-P124)	− 3
			BUR (P124 and P125)	
			OF, IRW, FCM (P126)	
			BW (P120 and P126)	

### Behavioral home cage assessment

#### Saccharin preference

In the PhenoTyper home cage, we assessed anhedonia-associated behavior successively over 4 days with the saccharin preference test. The PhenoTyper home cage provided a wall with two water bottles, which were filled with 200 g tap water on the first and third day. On day 2 and 4, animals had access to a water bottle containing 200 g of a 0.1% saccharin solution (Aldrich Saccharin ≥ 98%, Sigma-Aldrich Chemie GmbH, Taufkirchen, Germany) and a water bottle filled with 200 g tap water. To exclude a possible side preference of the animals, the side of the bottle containing the saccharin solution on the cage wall was switched from the right on day 2 to the left on day 4. The respective consumption over 24 h was measured and analysis was carried out as defined in a protocol by Klein et al.^62^. Data from one male prepubescent experimental unit (n = 2) was excluded in the analysis as one of the water bottles leaked on one of the testing days.

#### Burrowing

Burrowing performance was evaluated on two consecutive days, and analysis was carried out for the respective light and dark phases. In the afternoon, 2 h prior to the dark phase, an empty water bottle (length: 20 cm, diameter of the bottleneck: 3.5 cm; Zoonlab GmbH, Castrop-Rauxel, Germany) filled with 200 g ± 1 g food pellets (Ssniff Spezialdiäten GmbH, Soest, Germany), was placed in the PhenoTyper home cage. After 120 min, the weight of the bottle with the remaining pellets was measured. After removal of the distributed pellets in the animals’ home cage, the bottle was replaced overnight. On the next day, immediately after the dark phase, the weight of the bottle with the remaining pellets was measured again.

#### Nest building

We investigated nest building performance in the PhenoTyper home cage, assessing the complexity and shape of the nest over 6 days. On the first testing day, animals were offered two pressed cotton squares per PhenoTyper home cage. Pictures of the nest were taken on a daily basis in the morning, including a top-down view and two side views at an angle of 90 and approximately 45 degrees. Several protocols for the assessment of nestbuilding performance in mice have been established^37,63,64^. For the image-based evaluation of the nest complexity, we applied a slightly modified version of a protocol reported by Jirkof et al.^37^. For detailed information about the applied scoring scheme, see Supplementary information. The scoring of the images was carried out by a person who was blinded for the group allocation.

#### Voluntary wheel running

We assessed the development of voluntary wheel running activity in the PhenoTyper home cage over four consecutive days. Thereby, we evaluated the time necessary to adapt to the running wheel and the progressive increase in running activity. Animals were exposed to the PhenoTyper home cage providing a 24 h per day freely accessible running wheel (diameter: 15 cm, width: 7 cm; PhenoWheel, Noldus, Wageningen, the Netherlands). Analysis of voluntary wheel running activity was performed with the integrated software EthoVision XT 15 (EthoVision XT, RRID:SCR_000441), based on the counts registered per minute.

### Open field test

At the end of each testing week, exploratory behavior and locomotor activity were analyzed in the open field test. For habituation, animals were transferred to the testing room at least 30 min prior to the experiment. The animal was placed in a circular shaped open field (diameter: 60 cm; lighting condition: 20 lx), with its head facing the wall, 10 cm apart from the wall. The open field tests for each experimental unit (n = 2) were conducted in parallel, placing the two animals in its respective arenas simultaneously. Tracking duration lasted 15 min. Open fields were cleaned with 0.1% acetic acid after each trial. Male animals were tested prior to female animals. Analysis was carried out using the tracking software EthoVision XT 8.5 (EthoVision XT, RRID:SCR_000441). Additionally, rearing frequency was counted manually by a person unaware of the group allocation.

### Body weight

The development of the body weight of the animals was measured on the first day on Fridays and the last day on Tuesdays of the testing week.

### Irwin score

General condition of the mice was evaluated on the sixth day of the testing week, based on the traditional Irwin scoring system^55^, which provides information about general behavioral, neurological, and vegetative changes. We applied a slightly modified Irwin scale, divided into three consecutive parts (home cage, Open field, fresh single cage), including rectal body temperature measurement. Detailed information on the applied scoring system is provided in the Supplementary file.

### Fecal corticosterone metabolites

Samples for the assessment of fecal corticosterone metabolite concentrations were obtained from animals (n = 200) after the Open field test on the last testing day. For detailed information on sampling, processing and analysis, see the Supplementary file.

### Statistics

Statistical analysis of group differences was carried out using GraphPad Prism 5.04 for Windows (GraphPad Prism Software, San Diego, USA). We evaluated group differences by two-way analysis of variance (ANOVA), focusing on the two factors age phase and sex, followed by Bonferroni multiple comparison test, or one-way ANOVA, followed by Bonferroni multiple comparison test. Where indicated we determined group differences based on a model of linear regression. A p value < 0.05 was considered statistically significant. For nest complexity scores the median is shown. All other data are expressed as mean ± SEM.

## Supplementary Information

Supplementary Information.

## Data Availability

All datasets used for the analysis will be made available in the severity assessment repository of the DFG research unit 2591: https://for.severity-assessment.de.
