# Supplementary material for: Development of behavioral patterns in young C57BL/6J mice: a home cage-based study
Source: Sci Rep. 2022 Feb 15;12:2550. doi: 10.1038/s41598-022-06395-1 (PMC8847349; doi:10.1038/s41598-022-06395-1)
Supplement: Supplementary file 1 — Supplementary Information. [file 41598_2022_6395_MOESM1_ESM.pdf]

Development of behavioral patterns in young C57BL/6J mice: a home cage-based study

Maria Reiber, Ines Koska, Claudia Pace, Katharina Schönhoff, Lara von Schumann, Rupert Palme and Heidrun Potschka

Irwin Score

We applied a slightly modified Irwin scale, divided into three consecutive parts:

1. Observation in the PhenoTyper home cages,

2. Observation during the Open field test,

3. Observation and handling in fresh Makrolon type II open cages (Ehret GmbH & Co. KG, Emmendingen, Germany), enriched with bedding material (Lignocel Select, J. Rettenmaier & Söhne GmbH & Co. KG, Rosenberg, Germany).

Irwin scoring was carried out from the least invasive parameters assessed in the home cages to the more interfering, handling-associated parameters. An overview of the parameters is provided below.

Parameters assessed during observation in the PhenoTyper home cages:

1) Body posture
-2 flat, lying position, no muscle tone
-1 partly preserved muscle tone, occasional postural corrections
0 normal
+1 rigid, tense posture (e.g. opisthotonos)
+2 animals show no resting position, no analysis possible

2) Position of pelvis
-1 flattened
0 barely altered
+1 elevated

3) Height of tail
-1 tail repeatedly touching the ground during movement
0 normal tail position
+1 tail erected (max. 90 ° angle) during periods of resting and activity, possibly beating of tail
+2 tail erected (Straub-phenomenon)

4) Limb rotation (outward)
0 not present
+1 minimal
+2 clear

5) Spontaneous locomotor activity
-2 no activity
-1 reduced movement, occasional grooming
0 normal activity
+1 increased, possibly powerful, possibly angular movement
+2 restless motion type

6) Ptosis
0 eyelids open
+1 eyelid partly open
+2 eyelid closed

7) Exophthalmoses
0 no exophthalmoses
+1 minimal
+2 clear

8) Lacrimation
0 not present
+2 present

9) Hypersalivation
0 not present
+1 present

10) Respiration
+2 agonal respiration, acute respiratory distress
-1 reduced, irregular, tense respiration
0 normal respiration
+1 increased respiratory frequency
+2 increased respiratory rate is also present during resting periods

11) Piloerection
0 not present
+ 1 present: mild to moderate grade
+2 present: high grade

12) Ataxia
0 not present
+1 coordination problems
+2 loss of coordination

13) Stereotypies
0 not present
+2 present

14) Freezing – towards presence of observer
0 not present
+1 freezing slightly visible
+2 clear, abrupt freezing

15) Trembling/tremor
0 not present
+2 present

16) Convulsions
0 not present
+2 present

17) Seizures
0 not present
+2 present

18) Vocalization
0 not present
+1 occasional, spontaneous, quiet
+2 more frequent, spontaneous, louder

Parameters assessed during observation in the open field arenas:

1) Ataxia
0 not present
+1 coordination problems
+2 loss of coordination

2) Freezing – when animal is placed into the arena
0 not present
+1 freezing slightly visible
+2 clear, abrupt freezing

3) Trembling/tremor
0 not present
+2 present

4) Convulsions
0 not present
+2 present

5) Seizures
0 not present
+2 present

6) Vocalization
0 not present
+1 occasional, spontaneous, quiet
+2 more frequent, spontaneous, louder

Observation in fresh single cages and handling-associated parameters:

1) Trembling/tremor
0 not present
+2 present

2) Convulsions
0 not present
+2 present

3) Seizures
0 not present
+2 present

4) Vocalization
0 not present
+1 occasional, spontaneous, quiet
+2 more frequent, spontaneous, louder

5) Curiosity towards a presented object (pen)
-2 no reaction
-1 decreased curiosity
0 normal interest towards the presented object
+1 jerky pursuit, not adjusting to repeated withdrawal
+2 attack behavior

6) Touch response
-2 loss of response, no reaction even to increased stimuli
-1 repeated or increased stimulus provokes slow, reduced reaction (duck, evasion)
0 normal reaction to slight touching
+1 evasion of or ducking from slight touching
+2 flight over greater distance, attack behavior, vocalization to slight touching

7) Startle response
-2 no reaction
-1 delayed or decreased reaction
0 normal
+1 flight
+2 strong reaction, blepharospasm, laid-back ears, ducking on the ground or temporary freezing

8) Irritability - bite propensity(handling)
0 no irritability
+1 minimal
+2 clear

9) Tone of body (handling)
-2 completely limp
-1 reduced muscle tone
0 normal muscle tone
+1 increased muscle tone

+2 rigid

10) Abdominal wall tension (handling)
-1 reduced muscle tone
0 normal
+1 increased muscle tone

11) Urination (handling)
0 not present
+1 present

12) Defecation (handling)
0 not present
+1 present

13) Feces
-1 no or scarce amount of feces in the cage
0 character and amount of feces normal
+1 increased amount of feces
+2 almost liquid feces, diarrhea

14) Positional reflexes
Assessed only in case animal is continuously lying on the ground
-2 not present
-1 reduced
0 no alterations

Analysis of fecal corticosterone metabolites

The collection of fecal samples was carried out in the morning (7 to 11 a.m.) directly after the Open field paradigm. Feces of the mice (n=200) were collected in the Open field arenas, where the animals were placed and tested individually. In order to collect a sufficient amount of sample material, animals were then placed individually into Makrolon open cages type II (Ehret GmbH & Co. KG, Emmendingen, Germany), supplemented with bedding material (Lignocel Select, J. Rettenmaier & Söhne GmbH & Co. KG, Rosenberg, Germany), and feces were collected from these cages after two hours. The samples were stored frozen at -20°C. For processing, feces were dried and homogenized, and aliquots of 0.05 g were extracted with 1 ml of 80% methanol[1]. The samples were analyzed using a 5α-pregnane-3β,11β,21-triol-20-one enzyme immunoassay, which has been established and fully validated for the measurement of fecal corticosterone metabolites in mice[2,3].

Analysis of nest building behavior

For the image-based analysis of nest building behavior we applied the following scoring scheme: Score 1: Nestlets are almost not manipulated (> 90% are intact); Score 2: Nestlets are slightly manipulated (50-90% are intact); Score 3: nestlets are largely manipulated (50-90% are torn); Score 4: Flat nest (> 90% are torn, shreds are placed in one quarter of the cage, < 50% of the walls are higher than the mouse); Score 5: Nearly perfect nest (> 90% are torn, > 50% of the walls are higher than the mouse); Score 6: Perfect nest (> 90% are torn, > 90% of the walls are higher than the mouse).

Supplementary Figures

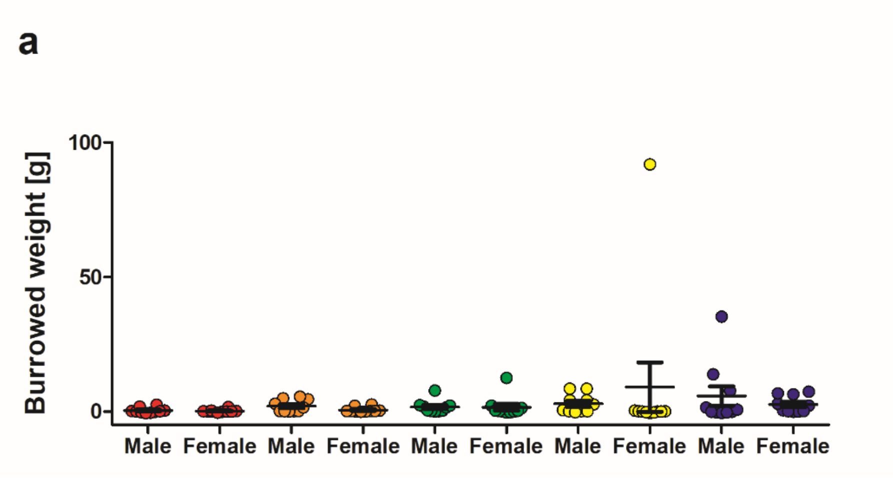

Supplementary Figure S1. Burrowing performance. In the first burrowing test session during the light phase **(a)**, a relatively low mean performance was observed among all age groups. Age- and sex-related differences were not detected (interaction p=0.6497, age phase p=0.3715, sex p=0.8856). Two-way ANOVA, followed by Bonferroni multiple comparison tests. * p<0.05. Colored dots refer to the respective age group (red P25, orange P36, green P50, yellow P65, blue P120). Error bars indicate the standard error of the mean (SEM).

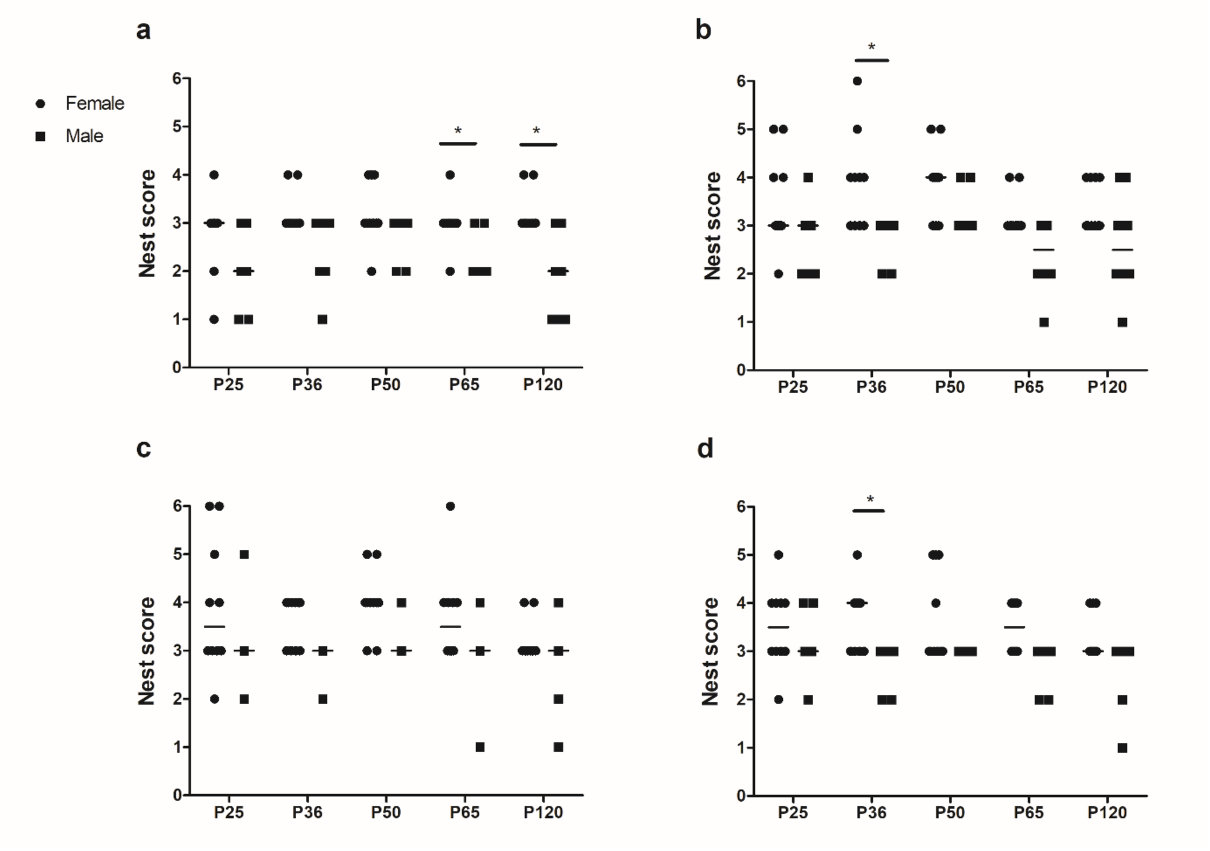

Supplementary Figure S2. Nest building performance and nest complexity. Differences in nest building performance between female and male mice were evident **(a)** on the second day (interaction p=0.2337, age phase p=0.0330, sex p<0.0001), **(b)** on the third day (interaction p=0.9074, age phase p=0.0189, sex p<0.0001), and **(d)** on the fifth day (interaction p=0.7770, age phase p=0.3940, sex p<0.0001) after offering new nesting material. On day 4 **(c)**, no sex-related differences were detected (interaction p=0.8689, age phase p=0.1472, sex p<0.0001). Two-way ANOVA, followed by Bonferroni multiple comparison tests. * p<0.05, median.

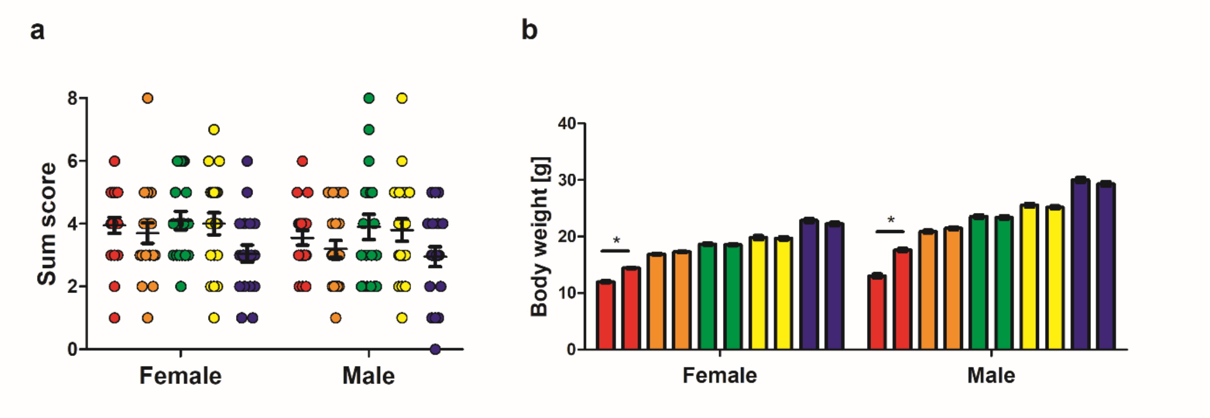

Supplementary Figure S3. Analysis of Irwin sum scores **(a)** revealed neither age- nor sex-dependent differences (interaction p=0.9681, age phase p=0.0115, sex p=0.1587). Comparison of body weights from start day (day 1, Friday, left bar) and final day (day 6, Thursday, right bar) within the age groups **(b)** indicated a pronounced body weight gain in prepubescent mice of both sexes (interaction p=0.0001, age phase p<0.0001, sex p<0.0001). Body weight differences between experimental start and end date were not observed in the other age groups. Two-way ANOVA, followed by Bonferroni multiple comparison tests. * p<0.05. Colored dots/bars refer to the respective age group (red P25, orange P36, green P50, yellow P65, blue P120). Error bars indicate the standard error of the mean (SEM).

Literature

1 Palme, R., Touma, C., Lepschy, M., Arias, N. & Dominchin, F. Steroid extraction: Get the best out of faecal samples. *Wiener tierärztliche Monatsschrift* **100**, 238-246 (2013).

2 Touma, C., Sachser, N., Möstl, E. & Palme, R. Effects of sex and time of day on metabolism and excretion of corticosterone in urine and feces of mice. *Gen Comp Endocrinol* **130**, 267-278, doi:10.1016/s0016-6480(02)00620-2 (2003).

3 Touma, C., Palme, R. & Sachser, N. Analyzing corticosterone metabolites in fecal samples of mice: a noninvasive technique to monitor stress hormones. *Horm Behav* **45**, 10-22, doi:10.1016/j.yhbeh.2003.07.002 (2004).
